# Anatomical success rate of pars plana vitrectomy for treatment of complex rhegmatogenous retinal detachment

**DOI:** 10.1186/s12886-016-0390-2

**Published:** 2016-12-09

**Authors:** Xhevat Lumi, Zala Lužnik, Goran Petrovski, Beáta Éva Petrovski, Marko Hawlina

**Affiliations:** 1 Eye Hospital, University Medical Centre Ljubljana, Grablovičeva 46, 1000 Ljubljana, Slovenia; 2 Department of Ophthalmology, Faculty of Medicine, Albert Szent-Györgyi Clinical Center, University of Szeged, Korányi fasor 10-11, 6720 Szeged, Hungary; 3 Centre of Eye Research, Department of Ophthalmology, Oslo University Hospital, University of Oslo, Oslo, Norway; 4 Department of Public Health, Faculty of Medicine, University of Szeged, Dóm tér 10, 6720 Szeged, Hungary; 5 Health Services Research Centre, Akershus University Hospital, 1478 Lørenskog, Norway, Institute of Clinical Medicine, Campus Ahus, University of Oslo, Oslo, Norway

**Keywords:** Rhegmatogenous retinal detachment, Pars plana vitrectomy, Proliferative vitreoretinopathy, Pseudophakia, Axial length

## Abstract

**Background:**

Pars plana vitrectomy (PPV) is preferred surgical procedure for the management of complex rhegmatogenous retinal detachment (RRD). The purpose of this study was to evaluate the anatomical results of primary PPV for the treatment of primary complex RRD and to determine the influence of lens status, tamponading agent, preoperative proliferative vitreoretinopathy (PVR) and axial length (AL) of the eye upon the anatomical outcome.

**Methods:**

A retrospective consecutive chart analysis was performed on 117 eyes from 117 patients with complex RRD managed with PPV. Fifty-nine eyes were phakic and 58 pseudophakic eyes. All patients had a minimum follow-up period of 12 months. Eyes were classified into groups using independent variables (first classification based upon lens status and tamponade used, second classification based upon lens and PVR status and third classification based upon AL of the eye). The groups were compared for anatomical outcomes (dependent variables) using nonparametric- or, in case of normally distributed data, parametric- statistical tests.

**Results:**

Retinal reattachment rate in phakic eyes was 94.9% compared to 93.1% in pseudophakic, with no statistically significant difference between the two. The overall retinal reattachment rate with single surgery was 94.0%. Final reattachment rate was 97.4%. In case of established PVR ≥ C1, the reattachment rate was not statistically different (92.6%) from eyes with no PVR (91.1%) irrespective of lens status. A statistically significant difference was found between redetachment rates only between phakic eyes with gas tamponade compared to silicon oil (SO) (*p* = 0.001). Reattachment rate proved to be similar in both AL groups (≤24 mm and > 24 mm).

**Conclusions:**

High anatomical success rate of primary vitrectomy for complex RRD with either gas or SO tamponade was achieved in phakic as well as pseudophakic eyes irrespective of AL of the eye.

## Background

Rhegmatogenous retinal detachment (RRD) is the most common vision-threatening retinal condition requiring urgent care [1]. There are three critical pre-conditions for the development of RRD: liquefied vitreous, tractional forces that produce a retinal break, and fluid access into the subretinal space through the retinal break [2, 3]. Several major risk factors that might predispose to RRD have been described [4]. Cataract surgery has been identified as one of the main predisposing risk factors [5].

RRD can be clinically classified as simple or complex detachment. In simple RRD, the retinal detachment is localized to a single, small retinal tear or hole at the retinal periphery accompanied by good visibility of the fundus [6]. In complex RRD, the detachment is partial, subtotal or total with a giant retinal tear, retinal dialysis, multiple retinal breaks, posterior breaks and also, it can be associated with vitreous hemorrhage, ocular trauma and proliferative vitreoretinopathy (PVR) [6, 7].

The aim of RRD treatment is identification and localization of retinal tears and their closure, as well as removing any traction on the edges of the tear [3]. Standard treatment modalities include: pneumatic retinopexy, scleral buckling and vitrectomy [8]. The appropriate surgical approach is chosen according to the complexity of detachment, age of the patient and the surgeon’s preference. Pneumatic retinopexy and scleral buckling surgery are the methods of choice for uncomplicated, simple RRD [9]. In case of complex detachments, pars plana vitrectomy (PPV) is indicated [9].

PPV has several advantages compared to the scleral buckling technique [10]. In phakic eyes, however, access to the vitreous base is technically difficult, making PPV in such cases challenging, possibly being the reason for having higher incidence of redetachment in these patients [11, 12]. In pseudophakic or aphakic eyes, on the other hand, a direct approach to the vitreous base may facilitate shaving of the vitreous base and also recognition and management of intraoperative vitreoretinal pathology [10]. This is the meaningful reason for many retinal surgeons to prefer a combined surgical technique (PPV with phacoemulsification and intraocular lens (IOL) implantation) [13]. Combined phacovitrectomy in patients with RRD has its advantages, but there are also doubts. Reports of several obstacles to complete vitreous base shaving, including complicated cataract surgery with residual lens cortex and inadequate pupillary dilation, as well as postoperative complications such as fibrinous uveitis, iris/IOL capture, intraocular pressure (IOP) rise and posterior capsular opacification exist in literature [13, 14]. Several concerns about the combined approach of PPV and phacoemulsification in RRD have been mainly the inaccuracy in calculating IOL power with significant myopic shift and also presence of unstable anterior chamber during peripheral scleral indentation [15–18].

Preoperative PVR (grade C) [19] has also been identified as one of the main risk factors for surgical failure following vitrectomy for RRD [20]. There are reports that combination of vitrectomy with scleral buckle in high risk patients for postoperative PVR causes significantly higher rates of anatomical success compared to PPV alone [21].

The end of the PPV surgical procedure for RRD always involved some sort of tamponade being employed [3]. The most often used tamponade for complex RRDs are perfluoropropane gas (C_3_F_8_) and silicone oil (SO) [22]. SO is frequently used in patients with complex RRDs, when retinectomies are unavoidable, in patients having lost the fellow eye or in patients unable to comply with postoperative head positioning [10]. There are several different reports on the outcomes of surgery and reattachment rates for SO and intraocular gas tamponade in a heterogeneous groups of patients with RRD and the differing surgical approaches. Because of the high heterogeneity, these results are not possible to compare [23–25].

Nevertheless, the literature contains few reports on whether the axial length (AL) of the eye has any impact on the anatomical results of PPV for RRD, myopia and high myopia have been characterized as factors that are significantly associated with either anatomical or functional failure of PPV for RRD. For this reason we decided to evaluate the impact of AL of the eye on the results of surgery in the studied group of patients [26–29].

Although refractive error of the eye is multifactorial where corneal power, lens power and AL play crucial role, studies of AL by ultrasound have shown that AL is most important factor in the development of myopia [30]. As AL shows a bimodal distribution in an adult myopic population with a first peak around the AL of 24 mm and second peak at AL around 30 mm, we classified our patients in groups with non-myopic AL (up to 24 mm) and with myopic AL (over 24 mm) [31].

In our surgical approach, we meticulousely clean the vitreous base without removing the crystalline lens. None of our patients included in the study period received scleral buckle or encircling band. As lens status, tamponading agent, presence of preoperative PVR and AL might influence the anatomical outcomes of primary PPV, this retrospective analysis evaluates the influence these factors have on the primary anatomical success rate of vitrectomy for complex RRD.

## Methods

### Study design

The study design adhered to the tenets of the Declaration of Helsinki; written informed consent from all patients was obtained before the surgical procedure as well as approval from the National Medical Ethics Committee of the Republic of Slovenia.

The patients were sampled from the registry in the Eye Hospital, University Medical Centre Ljubljana, Slovenia. A retrospective study of the medical records of 117 eyes of 117 patients that underwent primary small-gauge PPV for complex RRD was carried out. The surgical procedures were performed consecutively between September, 2011 and September, 2013 by one experienced retinal surgeon (XL).

For the purpose of the study, only patients undergoing vitrectomy due to complex RRD were selected [6]. Patients with subtotal or total RRD with a giant retinal tear, retinal dialysis, multiple retinal breaks, posterior breaks, RRD with vitreous hemorrhage, RRD after penetrating eye injury and retinal detachments with preoperative PVR grade C1 or higher, were included in the study. PVR stage was graded according to the updated classification of Retina Society Terminology Committee (1991) [19].

In eyes with clear optic media and macula on retinal detachment AL measurement was provided by IOL Master Optical Biometer (IOL Master, Carl Zeiss Meditec AG). In eyes with opaque optical media or macula – off detachment A-scan ultrasound biometry was performed using 10 MHz frequency probe. For the mean AL value, six AL measurements were averaged for each eye.

Patients with uncomplicated RRD were otherwise managed by scleral buckling surgery and/or pneumatic retinopexy. Excluded from the study were patients younger than 16 years of age, aphakic, patients having proliferative diabetic retinopathy or retinal dystrophies and 3 patients with incomplete follow-up period.

### Surgical procedure

In all cases, 23 or 25 Gauge PPV was performed using a noncontact wide-angle viewing system. After receiving informed consent, surgery was performed either under general or local anaesthesia. Trocars were placed in a way that allows peripheral vitrectomy to be performed without touching the lens, and also switching between the 3 entry sites, if necessary. The arrangement of sclerotomy sites in combination with 29 Gauge chandelier endoilumination, and bimanual work allowed for safe shaving of the peripheral vitreous to be carried out. Endolaser photocoagulation using curved probe was applied either around the retinal tear or 360° to the vitreous base. Patients received non-expansile perfluoro-n-octane (C_3_F_8_) diluted in air (10–15%) or SO (2000 Centistokes) tamponade at the end of the surgery. SO was instilled in cases with extended retinectomies or giant tears (larger than 6 clock hours), in patients with RRD in the only functional eye and in those who were unable to stay in prone position after surgery. Patients were examined postoperatively and followed for at least 12 months after the last surgery.

PPV alone was performed in 117 cases. Only in 3 cases with dense cataract a combined surgical technique was performed (PPV with phacoemulsification and IOL implantation). For statistical purposes, these 3 cases were included in the pseudophakic group. At the three-year follow-up period, 35 phakic patients had additional cataract surgery (phacoemulsification with IOL implantation).

Surgery was considered successful only in cases when retina remained attached at one-year follow-up after a single procedure in eyes treated with PPV and gas tamponade or one year after oil removal in eyes with PPV and SO tamponade.

### Groups of patients

Eyes were first classified into groups according to the lens status (phakic, pseudophakic), and the tamponading agent used at the end of the surgery (gas or SO):

- Group 1 (G1) (*n* = 46): PPV in phakic eyes + gas tamponade
- Group 2 (G2) (*n* = 43): PPV in pseudophakic eyes + gas tamponade
- Group 3 (G3) (*n* = 13): PPV in phakic eyes + SO tamponade
- Group 4 (G4) (*n* = 15): PPV in pseudophakic eyes + SO tamponade.

The second classification was done according to the lens status and the presence or absence of preoperative PVR grade C1 or more:

- Group 5 (G5) (*n* = 43): PPV in phakic eye without preoperative PVR C1
- Group 6 (G6) (*n* = 48): PPV in pseudophakic eye without preoperative PVR C1
- Group 7 (G7) (*n* = 16): PPV in phakic eye with preoperative PVR ≥ C1
- Group 8 (G8) (*n* = 10): PPV in pseudophakic eye with preoperative PVR ≥ C1

Additionally, the eyes were classified according to their AL (*n* = 93):

- Group 9 (G9) (*n* = 40): PPV in eyes with AL ≤ 24 mm
- Group 10 (G10) (*n* = 53): PPV in eyes with AL > 24 mm

### Statistical analysis

Qualitative variables were categorized, whereas quantitative data were presented as mean ± standard deviation (SD), median and/or range. Demographic factors investigated in the tested population included age, gender, duration of symptoms, preoperative visual acuity (BCVA, presented as logMAR), preoperative PVR status, preoperative lens status, retinal redetachment rate and AL.

Eyes were classified into groups using independent variables (first classification regarding the lens status and tamponade used; second classification regarding lens and PVR status; third classification regarding AL as shown above). The groups were compared for anatomical outcomes (dependent variables) using non-parametric statistical test or parametric statistical test in case of normally distributed data. Chi-square or Fisher’s exact test was used for categorical variables. Mann–Whitney *U* test and Kruskal-Wallis test were used as non-parametric methods to detect whether median of two or more groups are different. Statistical comparisons between the study groups were performed using one-way analysis of variance (ANOVA). The difference in anatomical outcomes (retinal redetachment rate – dichotomous variable) between the groups was evaluated by Fisher exact test. *P* values were considered statistically significant if *p* < 0.05. Statistical analysis was performed using the SPSS V20.0 (IBM, Corporation, Armonk, NY) and Stata/SE (version 11, StataCorp, College Station, TX) software.

## Results

### Demographic data

**Fig. 1:**
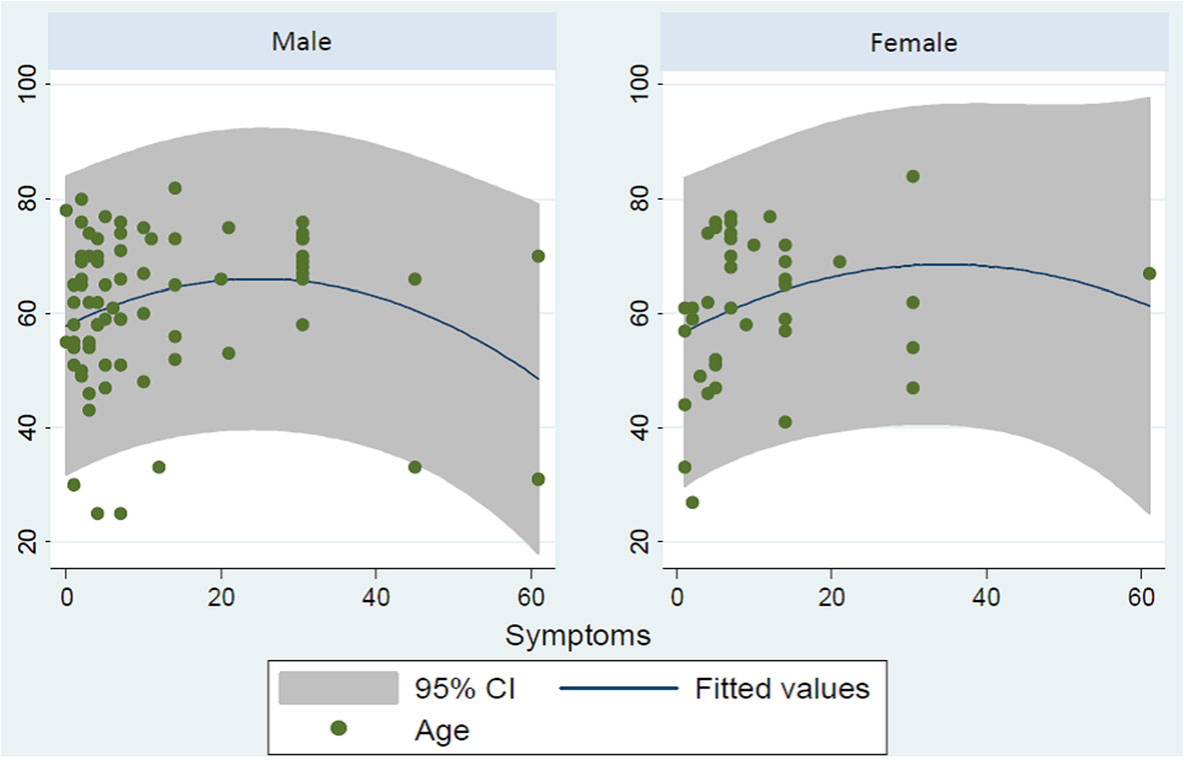
Scatter plot of duration of symptoms (days) and age (years) in the two studied gender

The retrospective study included a population of 117 eyes of 117 patients, characterized by a male predominance (77 males and 40 females; sex ratio: 1.9). A descriptive comparison between both gender groups in relation to age, duration of symptoms, median preoperative BCVA, preoperative lens status, preoperative PVR status and retinal reattachment rate 12-months after primary PPV was made. Between the gender groups, no statistically significant differences were found in terms of age and median duration of symptoms (Fig. 1).

There were also no statistically significant differences between gender groups in terms of preoperative BCVA and preoperative PVR status. The preoperative lens status showed statistically significant difference between the male and female group (*p* = 0.032). In the male group, 57.1% of the patients were pseudophakic, whereas in the female group 35.0% were pseudophakic. The anatomical success rates after primary PPV during the 12-month follow-up were similar in both gender groups without statistically significant difference (males: 94.8%; females: 92.5%; *p* = 0.7) (Table 1).

**Table 1:** General and ocular characteristics of patients included in the study. Descriptive statistics of numerical variables for both gender groups in relation to demographic profile

Factors Studied	All patients (*n* = 117 eyes)	Gender (sex ratio = 1.9)		*p*
		Male (*n* = 77)	Female (*n* = 40)	
BCVA preop (logMAR) (median, range)	1.3 (0–3)	1.0 (0–3)	2.0 (0–3)	0.25^a^
Lens status (n, %)				
Phakic	59 (50.4%)	33 (42.9%)	26 (65.0%)	0.032^b^*
Pseudophakic	58 (49.6%)	44 (57.1%)	14 (35.0%)	
PVR status				
PVR < grade C1 (n, %)	91 (77.8%)	62 (80.8%)	29 (72.5%)	0.3^b^
PVR ≥ grade C1 (n, %)	26 (22.2%)	15 (19.5%)	11 (27.5%)	
Reattachment rate (n, %)	110/117 (94%)	73/77 (94.8%)	17/40 (92.5%)	0.7^b^

*p < 0.05 was considered statistically significant. ^a^Mann–Whitney *U* test; ^b^Chi-Square test

### Effect of lens status and tamponading agent used (C_3_F_8_ vs. SO)

The influence of preoperative lens status on the anatomical outcomes of primary PPV for RRD are presented in Tables 2 and 3. In phakic eyes, the anatomical success rate at 12 months after surgery was 94.9% (56/59) compared to 93.1% in the pseudophakic eyes (54/58) irrespective of the tamponade being used (*p* = 0.7) (Table 2). The anatomical success rate with retinal reattachment 1 year after a single surgery or 1 year after SO removal was achieved in 94.0% of the cases (110/117 patients, Table 1). From the 7 patients with retinal redetachment, successful anatomical result was achieved with one reoperation in 4 patients, while in 3 patients, a decision was made to leave permanently the SO tamponade. The overall anatomical success rate was 97.4% (114/117).

**Table 2:** Effect of lens status (irrespective of the tamponade use) on the 12-month postsurgical retinal reattachment rate following PPV

Lens status	Phakic (*n* = 59 eyes)	Pseudophakic (*n* = 58 eyes)	*p*
Reattachment rate (%)	94.9% (56/59)	93.1% (54/58)	0.7

**Table 3:** Comparison between the studied groups in relation to demographic profile, history of symptoms, visual acuity and PVR status

Variables	Groups				*p*
	G1 (Phakic + gas) (*n* = 46)	G2 (Pseudophakic + gas) (*n* = 43)	G3(Phakic + SO) (*n* = 13)	G4 (Pseudophakic + SO) (*n* = 15)	
Male gender (n,%)	25/46 (54.7%)	32/43 (74.4%)	8/13 (61.5%)	12/15 (80.0%)	0.14^a^
Age (mean ± SD)	58.5 ± 13.5	62.7 ± 10.2	51.6 ± 17.8	70.7 ± 9.5	0.001^c*^
Duration of symptoms (days) (median, range)	7 (0–185)	7 (1–256)	7 (1–730)	6 (0–230)	0.9^d^
Preop BCVA (in logMAR) (median, range)	1.5 (0.05–3)	0.52 (0–3)	2 (0–3)	3 (0.2–3)	0.03^d*^
Preoperative PVR (n, %)	11/46 (23.9%)	4/43 (9.3%)	5/13 (38.5%)	6/15 (40.0%)	0.03^a*^
Reattachment rate (n,%)	100% (46/46)	95.3% (41/43)	76.9% (10/13)	86.7% (13/15)	0.011^b*^
Preoperative IOP (mean ± SD, range)	14.8 ± 4.7 (0–26)	14.2 ± 4.7 (5–24)	14.7 ± 3.2 (10–21)	10.9 ± 5.9 (0–21)	0.064^c^
Postoperative IOP (mean ± SD, range)	16.1 ± 4.0 (10–27)	14.8 ± 3.2 (9–25)	16.5 ± 7.0 (4–27)	13.8 ± 3.3 (9–19)	0.152^c^

*p < 0.05 was considered statistically significant, ^a^Pearson Chi-Square; ^b^Fisher’s exact test, ^c^ANOVA test; ^d^Kruskal–Wallis test

The comparison of the baseline characteristics including gender, age, median duration of symptoms, median preoperative visual acuity (logMAR), preoperative PVR status, preoperative and postoperative IOP and reattachment rate among the 4 evaluated groups (G1-G4) is shown in Table 3. The groups were balanced for gender, median duration of symptoms, pre- and post-operative IOP, and showed statistically significant difference in regards to preoperative visual acuity, age and PVR status. The lowest preoperative visual acuity (BCVA) was observed in group G4 (logMAR = 3), while higher in group G2 (logMAR = 0.52). In the same two groups, the lowest (G2 = 9.3%) and the highest (G4 = 40%) rate of preoperative PVR was also observed (Table 3).

Mean age was higher in group G4 and lowest in G3. Higher preoperative PVR rate was found in the groups treated with SO tamponade: G3 and G4 (38.5 and 40%, respectively). When the retinal reattachment rates were compared based upon the tamponade used, the highest rate was observed in group G1 (100%), followed by G2 (95.3%), G4 (86.7%) and G3 (76.9%), the difference between the groups being statistically significant (*p*  = 0.011). The preoperative and postoperative IOP values, on the other hand, showed no significant difference (*p* = 0.064 and *p* = 0.152 respectively, Table 3).

**Fig. 2:**
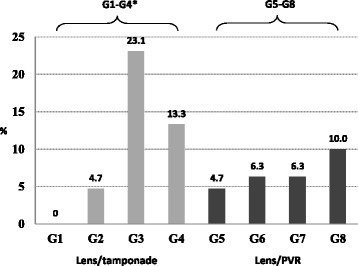
Comparison between tested groups for anatomical outcome (redetachment rate). *p < 0.05 was considered statistically significant. Fisher’s exact test

Anatomical outcome differed in the treatment groups (*p* = 0.011). Retinal redetachment rate was significantly higher in the group with phakic eyes after SO tamponade (G3), compared to phakic eyes with gas tamponade (G1) (*p* = 0.001); while the difference between group G3 and groups G2 and G4 (pseudophakic eyes with gas and SO tamponade, respectively) was not significant (Table 4, Fig. 2).

**Table 4:** Comparison between tested groups for anatomical outcome (redetachment rate)

Groups	Anatomical outcome (Redetachment rate %)
G1 (Phakic + gas; *n* = 46) vs G2 (Pseudophakic + gas; *n* = 43)	0% vs 4.7%; (*p* = 0.15)
G3 (Phakic + SO; *n* = 13) vs G4 (Pseudophakic + SO; *n* = 15)	23.1% vs 13.3%; (*p* = 0.5)
G1 (Phakic + gas; *n* = 46) vs G3 (Phakic + SO; *n* = 13)	0% vs 23.1%; ﻿(*p* = 0.001*)
G2 (Pseudophakic + gas; *n* = 43) vs G4 (Pseudophakic + SO; *n* = 15)	4.7% vs 13.3%; (*p* = 0.26)
G1-G4	*p* = 0.011*

*p < 0.05 was considered statistically significant. Fisher’s exact test

### Effect of Lens and preoperative PVR status

The anatomical outcome of primary PPV depending on the lens status and presence of preoperative PVR status are presented in Table 5 (groups G5-G8). The reattachment rates were: G5 = 95.3%, G6 = 93.7%, G7 = 93.8% and G8 = 90%, respectively. Although in phakic eyes (irrespective of PVR status) the redetachment rate was lower, the difference between the groups was not statistically significant (*p* = 0.9). Male gender was predominant in the three groups except in group G7. More often SO tamponade was used in the groups with preoperative PVR ≥ C1. The age significantly differed between the groups (*p* = 0.002). The mean age of patients from groups with phakic eyes was lower than patients in pseudophakic groups. The postoperative IOP showed also significant difference between the study groups (*p* = 0.02).

**Table 5:** Surgical details of the groups categorized according to the preoperative lens status and preoperative PVR status

Variables	G5 (Phakic + no PVR) (*n* = 43)	G6 (Pseudophakic + no PVR) (*n* = 48)	G7 (Phakic + PVR) (*n* = 16)	G8 (Pseudophakic + PVR) (*n* = 10)	*p*
Reattachment rate (%, n)	95.3% (41/43)	93.7% (45/48)	93.8% (15/16)	90.0% (9/10)	0.9^b^
Age (mean ± SD)	55.4 ± 14.9	63.9 ± 11.0	61.3 ± 13.6	69.6 ± 6.5	0.002^d*^
Duration of symptoms (days) (median, range)	5 (0–185)	7 (1–256)	14 (2–730)	6.5 (0–230)	0.075^c^
Male Gender (n, %)	26/43 (60.5%)	36/48 (75.0%)	7/16 (43.8%)	8/10 (80.0%)	0.08^a^
Tamponade SO (n, %)	8/43 (18.6%)	9/48 (18.8%)	5/16 (31.2%)	6/10 (60.0%)	0.08^a^
Preoperative IOP (mean ± SD, range)	15.1 ± 4.5 (0–26)	13.8 ± 5.1 (2–24)	11.1 ± 4.0 (7–21)	11.6 ± 5.4 (0–17)	0.21^d^
Postoperative IOP (mean ± SD, range)	16.9 ± 4.8* (4–27)	14.5 ± 3.4* (9–25)	14.1 ± 3.9* (6–22)	14.8 ± 2.7 (11–18)	0.02^d*^

*p < 0.05 was considered statistically significant. ^a^Chi-Square, ^b^Fisher’s exact test; ^c^ Kruskal–Wallis test; ^d^ANOVA test

Comparison of the retinal redetachment rate between groups G5-G8 showed no significant difference (Table 6, Fig. 2).

**Table 6:** Comparison between groups in relation to the lens status and preoperative PVR on retinal redetachment rate

Groups	Anatomical outcome
G5 (Phakic + no PVR) (*n* = 43) vs G6 (Pseudophakic + no PVR) (*n* = 48)	*p* = 0.7
G7 (Phakic + PVR) (*n* = 16) vs G8 (Pseudophakic + PVR) (*n* = 10)	*p* = 0.9
G5 (Phakic + no PVR) (*n* = 43) vs G7 (Phakic + PVR) (*n* = 16)	*p* = 0.8
G6 (Pseudophakic + no PVR) (*n* = 48) vs G8 (Pseudophakic + PVR) (*n* = 10)	*p* = 0.6
G5-G8	*p* = 0.9

### Effect of AL upon redetachment rate

AL of the eye was measured preoperatively in the affected eye. Reliable data could be provided for 93 eyes of the 117 studied. The rate of redetachment was similar in both AL groups without a statistically significant difference (*p* = 0.9) (Table 7).

**Table 7:** The effect of axial length on the redetachment rate

No. of eyes	Total (*n* = 93)	Axial Length (mm)		*p*
		≤24 mm (*n* = 40)	>24 mm (*n* = 53)	
Redetachment (n,%)	10/93	3/40 (7.5%)	4/53 (7.5%)	0.9

## Discussion

The past decades have recorded a rise in RRD incidence [32]. This rise has partially been linked to the increase in number of pseudophakic RRDs [33]. In parallel, a rapid rise in popularity of primary vitrectomy for RRD has occurred due to the better intraoperative control of complicated RRD and avoidance of complications typically associated with scleral buckles [6, 34]. A better access to the vitreous base in pseudophakic eyes, allows for a better completion of the recommended complete shaving of the vitreous base compared to phakic eyes [14]. A reattachment rate of 97.78% from the cases undergoing a single PPV surgery in primary pseudophakic RRD has been observed in series by Stangos et al. [35].

Anatomical results of surgery for complex RRD are certainly lower. There are contradictory reports in the literature regarding anatomical outcome of PPV for complex RRD. There are also contradictory reports on the success rate in phakic and pseudophakic patients. However, recent advances in vitreoretinal surgery with the introduction of the use of chandelier lights, has allowed bimanual work with self-indentation to be implemented, while curved vitreous cutter and curved endolaser probes have provided the opportunity to remove the vitreous base and perform peripheral laser retinopexy without the need for clear lens removal, as well as allowed for more aggressive and near complete vitreous base shaving in phakic patients [36–39].

The present study evaluated the influence of preoperative lens status, tamponading agent, presence of preoperative PVR and AL of the eye upon the anatomical outcomes of PPV in patients with primary complex RRD. In this cohort of patients, a male predominance was present, with a higher pseudophakic rate observed in males. After classification into defined groups and normalization for age, gender and duration of symptoms, the pseudophakic patients were found to be on average older (groups G2, G4, G6 and G8), while the mean duration of symptoms before patients were referred to ophthalmologist ranged from 0 to 730 days.

In patients with gas tamponade being used, the lowest retinal redetachment rate was observed in phakic eyes (G1 = 0%, 0/47), although the difference with the pseudophakic group (G2 = 6.8%, 3/47) was not statistically significant. The difference in the redetachment rate, however, was significant between the phakic groups (gas vs. SO tamponade, or groups G1 vs. G3), with a higher rate being observed in the phakic group treated by SO tamponade. Such difference could not be detected between the pseudophakic groups G2 and G4. As SO is used in more complicated detachments with PVR, where peripheral retinectomies are unavoidable, the highest rate of preoperative PVR in Groups 3 and 4 was expected. The difference in retinal reattachment rate between the groups was statistically significant (*p* = 0.011), with a higher rate being found in phakic eyes with gas tamponade (G1 = 100%) and lowest in phakic eyes with SO tamponade (G3 = 76.9%).

The overall anatomical success rate with retinal reattachment 1 year after a single surgery or SO removal was 94%. Considering exclusion of the 3 patients with incomplete follow-up as a surgical failure, the corrected reattachment rate would be 91.6%. In phakic eyes, the anatomical success rate was 94.9% compared to 93.1% in pseudophakic eyes (irrespective of the tamponade being used) (*p* = 0.7). Retinal redetachment was observed in only 3 phakic and 2 pseudophakic eyes. The overall results obtained in the present study showed better trend in comparison to the study by Caiado et al., where 28–29% redetachment rate was reported for phakic eyes subjected to primary PPV with C_3_F_8_ or SO tamponade, although their results are not truly comparable to ours, due to being reported on cases with uncomplicated RRD [10]. We also show that high retinal reattachment rate can be achieved without combining PPV and scleral buckle. Kinori et al. reported single surgery success in 81.3 and 87.1% of noncomplex primary RRD managed either with PPV or in combination with scleral buckle, respectively [40].

High rate of retinal reattachment with a single surgery was shown in the series of Brazitikos et al. [41], with a 94% success rate of primary PPV being reported in uncomplicated pseudophakic RRD in a series of patients having PVR stage B or less [41]. Our results are comparable to those of Teke et al. [14] performed on a large series of 894 patients with complex RRD (PVR grade C) managed with PPV and SO tamponade and showing a 86.8% anatomical success rate. An important difference between the latter series and ours is that they used a larger study population and they excluded from the analysis all patients with retinal redetachment prior to SO removal, as well as patients who developed detachment during SO removal [14]. Due to such exclusion of patients, it is not possible to conclude the primary surgery success rate. Regler et al. reported an overall 79% final anatomic success rate of PPV for complex RRD [42], while a 87.8% success rate was reported in complicated RRD cases by Ozdek et al. [43].

An evaluation of the reattachment rate in eyes classified according to the lens and preoperative PVR status (second classification) was further made. The reattachment rate for the tested groups with PVR < C1 (G5 and G6) was 95.3 and 93.7%, respectively. The lowest reattachment rate was observed in pseudophakic patients with PVR ≥ C1 (G8 = 90%), although no statistically significant difference could be shown between the groups (*p* = 0.9). These results implicate that lens status and presence of preoperative PVR have very little impact on the anatomical outcome. Single surgery anatomical success rate of 60% and final rate of 93% has been reported in patients with PVR C1-D managed with PPV with or without lens removal in the series of Quiram et al. [44]. Final surgery outcomes were therefore attributed to the technique of radical anterior base dissection combined with lens removal [44].

In patients who had surgery for PVR, the redetachment rate was 27.6% after SO removal in a series by Jonas et al. [26]. This study also found a higher incidence of retinal redetachment after removal of SO in patients with incomplete vitreous base shaving [26]. From the Silicone Study Group, a higher retinal redetachment rate after SO removal in patients with PVR-C3 and higher has also been reported [45]. In addition, several reports about surgery outcomes from complex retinal detachment surgery using heavy SO have shown success rates ranging between 39 and 92%, with different number of surgeries being required and a high incidence of complications being reported [42, 46–49]. Mancino et al. recently reported 100 and 90.4% success rate of vitrectomy with inferior 180° peripheral retinectomy and SO tamponade in subgroups of patients with recurent retinal detachment and PVR grade C after failed primary scleral buckle surgery and PPV, respectively [50].

The present study reports no significant difference in the redetachment rate between eyes with AL up to 24 mm and long eyes with over 24 mm in AL, which is in concordance with the results obtained by Jonas et al. [26], although there have also been reports of 2.1 times higher retinal redetachment rate after SO removal in highly myopic eyes [14, 27, 51].

Although other studies have already shown high success rates of primary PPV, to the best of our knowledge, our current retrospective study is the first one to demonstrate similar anatomical success rates between phakic and pseudophakic eye groups, irrespective of the tamponade being used, with a low redetachment rate between groups. None of the patients in our study received scleral buckle or encircling band during PPV.

This study has some limitations. First, it is a retrospective study. Second, it was not randomized, which was attempted to overcome by using a large series of patients with long follow-up periods. It is not possible to generalize that one can always have the same anatomical outcome from surgery; these depend upon many factors which cannot be captured in one single retrospective study. Therefore, a further larger and prospectively designed study is necessary to confirm the present findings.

## Conclusions

High anatomical success rate of primary PPV with either perfluoro-n-octane gas or SO tamponade could be achieved in phakic as well as pseudophakic eyes. The redetachment rate was not statistically significant between phakic and pseudophakic eyes, irrespective of the tamponade being used, presence of preoperative PVR and AL of the eye.

## Abbreviations

AL: Axial length
BCVA: Best corrected visual acuity
IOL: Intraocular lens
IOP: Intraocular pressure
PPV: Pars plana vitrectomy
PVR: Proliferative vitreoretinopathy
RRD: Rhegmatogenous retinal detachment
SO: Silicon oil
